# Fluid-Attenuated Inversion Recovery Sequence with Fat Suppression for Assessment of Ankle Synovitis without Contrast Enhancement: Comparison with Contrast-Enhanced MRI

**DOI:** 10.3390/diagnostics13111960

**Published:** 2023-06-04

**Authors:** Ji Hee Kang, Sung Gyu Moon, Hong-Geun Jung, Eun Young Kwon

**Affiliations:** 1 Department of Radiology, Konkuk University Medical Center, Seoul 05030, Republic of Korea; 2 Department of Orthopedic Surgery, Konkuk University Medical Center, Seoul 05030, Republic of Korea; 3 Department of Radiology, Kyung Hee University Hospital at Gangdong, Seoul 05278, Republic of Korea

**Keywords:** fluid-attenuated inversion recovery sequence, synovitis imaging, ankle synovitis, magnetic resonance imaging

## Abstract

The purpose of this study was to investigate the feasibility of the fluid-attenuated inversion recovery sequence with fat suppression (FLAIR-FS) for the assessment of ankle synovitis without contrast enhancement. FLAIR-FS and contrast-enhanced, T1-weighted sequences (CE-T1) of 94 ankles were retrospectively reviewed by two radiologists. Grading of synovial visibility (four-point scale) and semi-quantitative scoring of synovial thickness (three-point scale) were performed in four compartments of the ankle in both sequences. Synovial visibility and thickness in FLAIR-FS and CE-T1 images were compared, and agreement between both sequences was assessed. Synovial visibility grades and synovial thickness scores for FLAIR-FS images were lower than those for CE-T1 images (reader 1, *p* = 0.016, *p* < 0.001; reader 2, *p* = 0.009, *p* < 0.001). Dichotomized synovial visibility grades (partial vs. full visibility) were not significantly different between both sequences. The agreement in synovial thickness scores between the FLAIR-FS and CE-T1 images was moderate to substantial (κ = 0.41–0.65). The interobserver agreement between the two readers was fair for synovial visibility (κ = 0.27–0.32) and moderate to substantial for synovial thickness (κ = 0.54–0.74). In conclusion, FLAIR-FS is a feasible MRI sequence for the evaluation of ankle synovitis without contrast enhancement.

## 1. Introduction

The synovium is a thin connective tissue lining the synovial joints [1,2]. Proinflammatory mediators produced in arthritis induce synovitis, histologically characterized by synovial hyperplasia and vascularization [3]. Synovitis is associated with pain and disease severity in osteoarthritis and rheumatoid arthritis [4,5,6]. In ankle joints, repetitive sprains can cause localized synovitis resulting in soft tissue impingement syndrome [7,8]. The exact localization of synovitis is required for surgical planning in soft tissue impingement syndrome. Hence, the evaluation of synovitis is clinically important.

Contrast-enhanced magnetic resonance imaging (MRI) is currently the reference standard for synovitis imaging owing to the direct visualization of enhanced synovial tissue distinguished from joint fluid [1]. However, intravenous administration of gadolinium-based contrast agents increases scanning time and cost, as well as the risk of allergic reactions, nephrogenic systemic fibrosis, and gadolinium deposition in the brain [9]. Therefore, there is growing interest in evaluating synovitis without contrast enhancement.

Fluid-attenuated inversion recovery sequence with fat suppression (FLAIR-FS) has been introduced as a potential non-enhanced MRI technique for synovitis assessment. Signals from both joint fluid and fat are suppressed by a combination of FLAIR and fat suppression, thereby improving the conspicuity of the synovium [10]. The feasibility of FLAIR-FS for the assessment of knee or hip synovitis has been demonstrated in previous studies [10,11,12]. However, to the best of our knowledge, no previous study has applied the FLAIR-FS to the assessment of ankle synovitis.

Therefore, the purpose of this study was to investigate the feasibility of FLAIR-FS for the assessment of ankle synovitis using contrast-enhanced MRI as the reference standard.

## 2. Materials and Methods

Our institutional review board approved this retrospective study and waived the requirement for informed consent.

### 2.1. Patients

**Figure 1:**
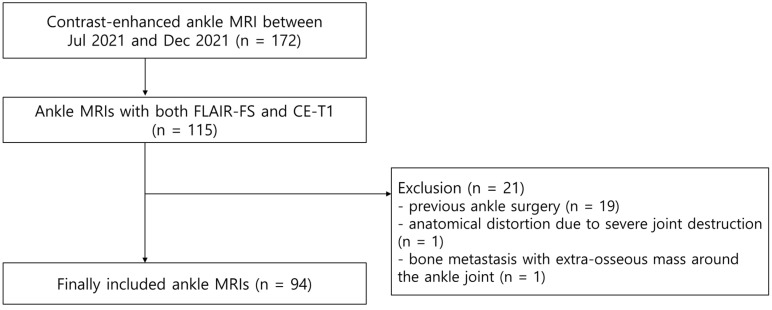
Flowchart shows the process used to enroll ankle MRIs.

A total of 172 consecutive patients underwent ankle MRI with contrast enhancement between July 2021 and December 2021 at our institution. Among them, 112 patients underwent 115 ankle MRIs, including both FLAIR-FS and contrast-enhanced, T1-weighted sequence (CE-T1) with fat suppression. The exclusion criteria were as follows: (a) previous ankle surgery (n = 19), (b) anatomical distortion due to severe joint destruction (n = 1), and (c) bone metastasis with an extra-osseous mass around the ankle joint (n = 1). Finally, 94 ankle MRIs from 92 patients (37 male, 55 female; mean age, 48.7 years; age range, 11–92 years) were enrolled (Figure 1). Fifty MRI scans were of the right ankle and 44 were of the left ankle. The reasons for ankle MRIs were chronic lateral ankle instability (n = 31), osteoarthritis (n = 18), osteochondral lesion (n = 16), sinus tarsi syndrome (n = 9), Achilles tendinitis (n = 4), nonspecific pain (n = 4), acute sprain (n = 3), osteonecrosis (n = 3), cellulitis (n = 1), foot drop (n = 1), tarsal coalition (n = 1), posterior tibialis tendinosis (n = 1), rheumatoid arthritis (n = 1), and stress fracture (n = 1).

### 2.2. Magnetic Resonance Imaging Protocol

The MRI was performed using two 3T units: Magnetom Vida (n = 45) or Magnetom Skyra (n = 49) with a dedicated 16-channel ankle coil (Siemens Healthcare, Erlangen, Germany). Routine ankle MRI sequences in our institution consisted of axial and coronal T2-weighted images, sagittal T1-weighted images, sagittal T2-weighted images with fat suppression, and axial, coronal, and sagittal 3D isotropic T2-weighted images. Axial FLAIR-FS and axial, coronal, and sagittal CE-T1 images with fat suppression were obtained after routine sequences. The detailed image parameters are presented in Table 1.

FLAIR-FS = fluid-attenuated inversion recovery sequence with fat suppression, and CE-T1 = contrast-enhanced, T1-weighted sequence.

To determine the optimal inversion time for FLAIR-FS, which effectively nulls joint fluid signals, several test scans were performed using inversion times between 2000 and 2200 ms. Consequently, an inversion time of 2100 ms was selected (Supplemental Table S1). Axial CE-T1 with fat suppression was performed 7 min after intravenous administration of contrast agent (gadoterate meglumine, Dotarem; Guerbet) at a dose of 0.2 mL/kg. For fat suppression, the “weak” mode of chemically selective fat suppression was applied in both FLAIR-FS and CE-T1 images.

### 2.3. Image Analysis

**Figure 2:**
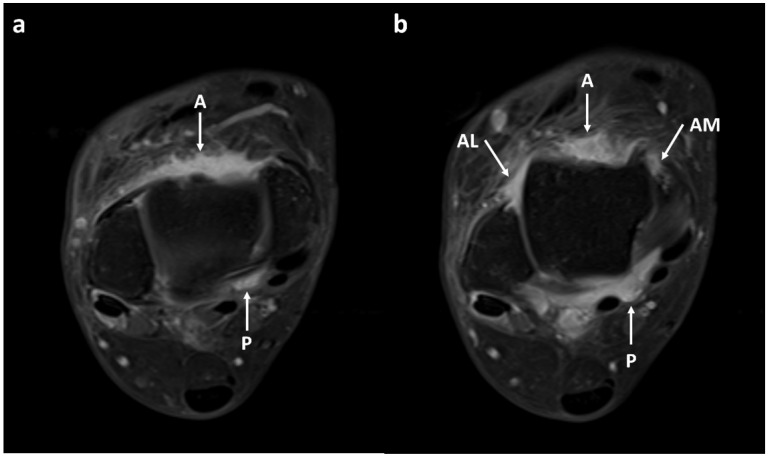
Axial contrast-enhanced, T1-weighted images showing synovial enhancement. The ankle joint was divided into four compartments: anterior recess (A), anteromedial gutter (AM), anterolateral gutter (AL), and posterior recess (P). (**a**) The anterior recess (A) was defined as the central portion of the recess between the anterior tibial plafond and the talar dome. The posterior recess (P) was defined as the posteromedial recess formed anteriorly by the medial malleolus and posterior tibiotalar ligament, laterally by the talar dome and posterior process of the talus, and peripherally by the flexor hallucis longus tendon and neurovascular bundle. (**b**) The anteromedial gutter (AM) was the space formed superficially by the joint capsule, laterally by the talus, medially by the medial malleolus, and inferiorly by the anterior tibiotalar ligament. The anterolateral gutter (AL) was the space formed medially by the tibia, laterally by the fibula, superiorly by the anteroinferior tibiofibular ligament, inferiorly by the calcaneofibular ligament, and anteriorly by the anterior talofibular ligament and joint capsule. Both readers assigned a synovial visibility grade of 4 (fully visible synovium with good tissue contrast) in all joint compartments. Reader 1 assigned a synovial thickness score of 1 (maximum thickness 2–4 mm) in the anteromedial gutter and 2 (maximum thickness ≥4 mm) in the other compartments. Reader 2 assigned a synovial thickness score of 2 in all joint compartments.

Two musculoskeletal radiologists (J.H.K. and S.G.M., each with 4 and 18 years of experience, respectively) independently reviewed the MRI images. They were blinded to the clinical information and diagnoses of the subjects. The ankle joints were divided into four compartments: anterior recess, anteromedial gutter, anterolateral gutter, and posterior recess (Figure 2) [7,13]. The anterior recess was defined as the central portion of the recess between the anterior tibial plafond and the talar dome. The anteromedial gutter was the space formed superficially by the joint capsule, laterally by the talus, medially by the medial malleolus, and inferiorly by the anterior tibiotalar ligament. The anterolateral gutter was the space formed medially by the tibia, laterally by the fibula, superiorly by the anteroinferior tibiofibular ligament, inferiorly by the calcaneofibular ligament, and anteriorly by the anterior talofibular ligament and joint capsule. The posterior recess was defined as the posteromedial recess formed anteriorly by the medial malleolus and posterior tibiotalar ligament, laterally by the talar dome and posterior process of the talus, and peripherally by the flexor hallucis longus tendon and neurovascular bundle. The posterolateral recess was excluded because it was difficult to measure synovial thickness consistently in that area owing to redundant joint capsule, joint communication with the flexor hallucis longus tendon sheath, and the os trigonum.

Synovial visibility and thickness in each compartment were assessed on FLAIR-FS and CE-T1 images, respectively. FLAIR-FS and CE-T1 images were analyzed independently and randomly. To assess intraobserver variability, both readers re-evaluated images at an 8-week interval, blinded to the results of the previous analysis. Synovial visibility was subjectively graded on a 4-point scale as follows: grade 1, no visible synovium; grade 2, partially visible synovium; grade 3, fully visible synovium with low tissue contrast; and grade 4, fully visible synovium with good tissue contrast [10]. To evaluate the agreement for synovial visibility between FLAIR-FS and CE-T1, grades 1 and 2 were classified as the partially visible group, and grades 3 and 4 were classified as the fully visible group. Synovial thickness was scored semi-quantitatively according to the maximum thickness of the synovium in each compartment as follows: score 0, < 2 mm; score 1, 2–4 mm; and score 2, ≥4 mm [14]. The scoring was performed by measuring the synovial thickness at the region where the synovium appeared thickest using PACS software (Centricity PACS 6.0, GE Healthcare). Synovitis was defined as synovial thickness greater than or equal to 2 mm (score 1 and 2 synovial thickness) on CE-T1 images [5,14]. Subsequently, the synovial thickness scores of the four compartments were summed to assess the severity of whole ankle synovitis. 

### 2.4. Statistical Analysis

The Wilcoxon signed-rank test was employed to compare synovial visibility and thickness on FLAIR-FS and CE-T1 images, regarding synovial visibility grade and synovial thickness scores as nonparametric measurements. The agreement between FLAIR-FS and CE-T1 images was assessed using the McNemar test for nominal variables and weighted kappa statistics for ordinal variables. Intraobserver and interobserver agreements were calculated using weighted kappa statistics for ordinal variables and intraclass correlation coefficients (ICC) for nonparametric measurements. Kappa values (κ) were interpreted based on the following criteria: κ < 0, poor agreement; 0 < κ ≤ 0.2, slight agreement; 0.2 < κ ≤0.4, fair agreement; 0.4 < κ ≤ 0.6, moderate agreement; 0.6 < κ ≤ 0.8, substantial agreement; 0.8 < κ ≤ 1, almost perfect agreement [15]. Intraclass correlation coefficients were interpreted as follows: ICC < 0.5, poor agreement; 0.5 ≤ ICC < 0.75, moderate agreement; 0.75 ≤ ICC < 0.90, good agreement; and 0.90 ≤ ICC, excellent agreement [16]. 

Statistical analyses were performed with SPSS 25.0 for Windows (SPSS Inc., Chicago, IL, USA) or MedCalc version 20.015 (MedCalc Software, Ostend, Belgium). A *p* value less than 0.05 was considered statistically significant.

## 3. Results

### 3.1. Patient Characteristics

Among the 94 ankles, synovitis was detected in 70 ankles (74.5%) by reader 1 and 68 ankles (72.3%) by reader 2. At the joint compartment level, 189 out of 376 joint compartments (50.2%) had synovitis on CE-T1 images. Synovitis was most common in the anterior recess (reader 1, 57/189, 30.2%; reader 2, 56/189, 29.6%), followed by the posterior recess (52/189, 27.5% for both readers), the anterolateral gutter (reader 1, 50/189, 26.5%; reader 2, 44/189, 23.3%), and the anteromedial gutter (reader 1, 30/189, 15.9%; reader 2, 37/189, 19.6%). In most cases, synovitis involved more than one joint compartment (reader 1, 56/70, 80%; reader 2, 57/68, 83.8%). 

### 3.2. Synovial Visibility on FLAIR-FS and CE-T1 Images

**Figure 3:**
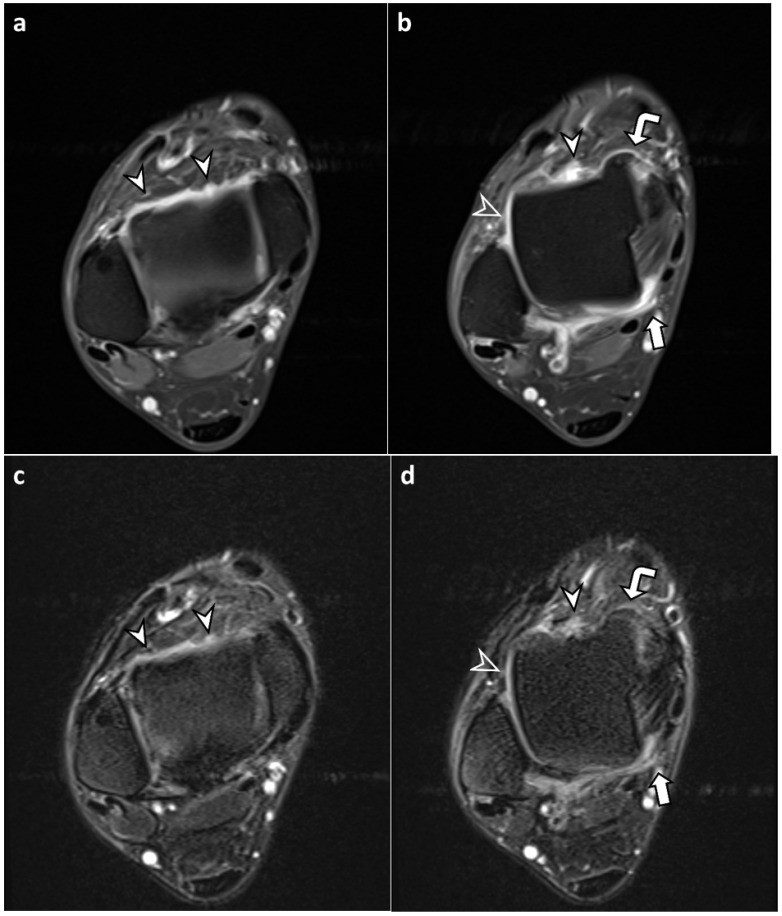
A 48-year-old male with chronic lateral ankle instability. (**a**,**b**) Axial contrast-enhanced, T1-weighted sequence (CE-T1) with fat suppression images reveal enhanced synovium in the anterior recess (arrowheads), anteromedial gutter (curved arrow), anterolateral gutter (open arrowhead), and posterior recess (arrow). Both readers assigned a synovial visibility grade of 4 (fully visible synovium with good tissue contrast) in all joint compartments. For synovial thickness score, reader 1 assigned 1 (maximum thickness 2–4 mm), 1, 0 (maximum thickness < 2 mm), and 2 (maximum thickness ≥4 mm) and reader 2 assigned 1, 2, 0, and 1 in the anterior recess, anteromedial gutter, anterolateral gutter, and posterior recess, respectively. (**c**,**d**) Corresponding axial fluid-attenuated inversion recovery sequence with fat suppression (FLAIR-FS) images in the same level reveal synovium showing hyperintense signal intensity, similar to CE-T1 images. Both readers assigned a synovial visibility grade of 4 in all joint compartments. For synovial thickness score, reader 1 assigned 1, 1, 0, and 2 and reader 2 assigned 1, 1, 0, and 1 in the anterior recess, anteromedial gutter, anterolateral gutter, and posterior recess, respectively.

**Figure 4:**
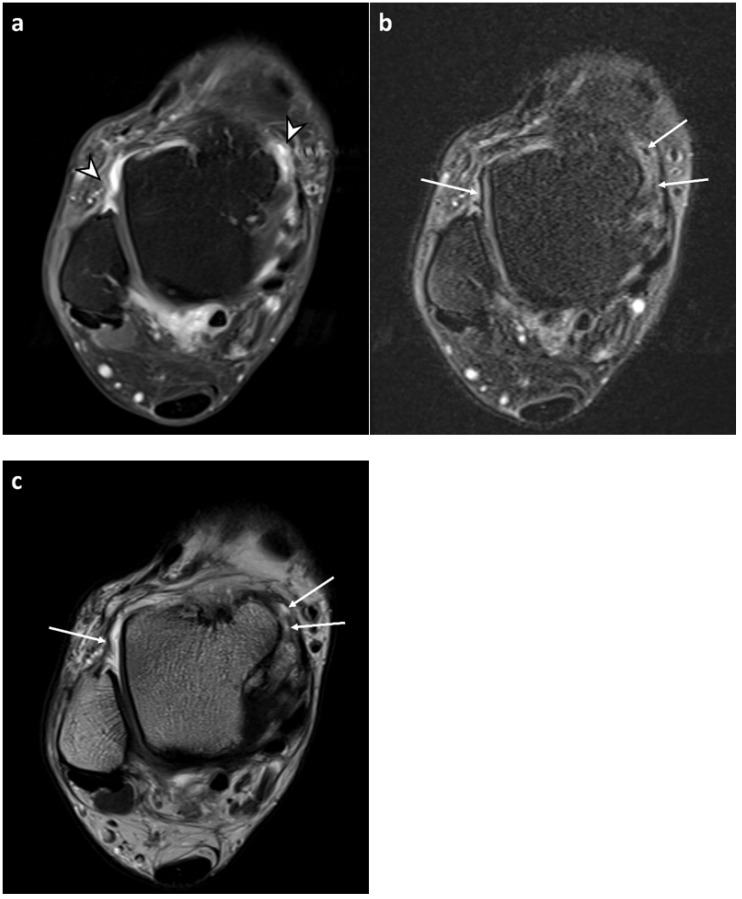
A 68-year-old male with osteoarthritis of the ankle joint. (**a**) Axial contrast-enhanced, T1-weighted sequence (CE-T1) with fat suppression image demonstrates enhanced synovium (arrowheads) with good tissue contrast. Both readers assigned a synovial visibility grade of 4 (fully visible synovium with good tissue contrast) in all joint compartments. For synovial thickness score, reader 1 assigned 2 (maximum thickness ≥ 4 mm), 1 (maximum thickness 2–4 mm), 1, and 2 and reader 2 assigned 2, 2, 2, and 2 in the anterior recess, anteromedial gutter, anterolateral gutter, and posterior recess, respectively. (**b**) Axial fluid-attenuated inversion recovery sequence with fat suppression (FLAIR-FS) image in the same level demonstrates dark signal intensity foci (arrows), suggesting suppressed joint fluid signals. Both readers assigned a synovial visibility grade of 4 in all joint compartments. For synovial thickness score, reader 1 assigned 2, 1, 0 (maximum thickness < 2 mm), and 1 and reader 2 assigned 1, 2, 0, and 1 in the anterior recess, anteromedial gutter, anterolateral gutter, and posterior recess, respectively. (**c**) Axial T2-weighted image in the same level shows joint fluid (arrows) at the location corresponding with dark signal intensity foci in FLAIR-FS image.

**Figure 5:**
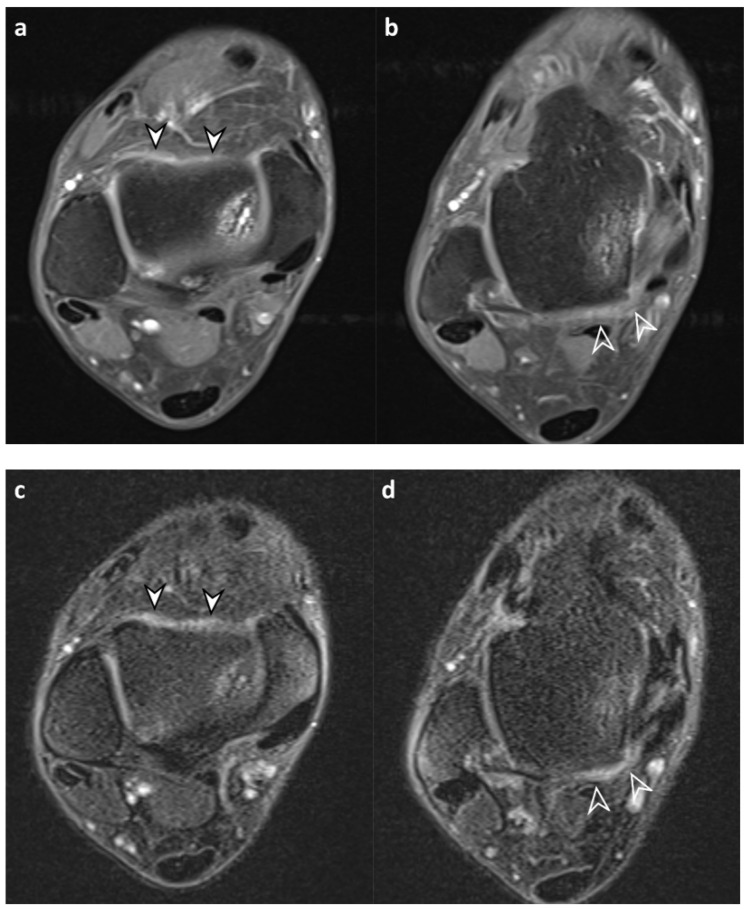
A 60-year-old male with osteochondral lesion of the talus. (**a**,**b**) Axial contrast-enhanced, T1-weighted sequence (CE-T1) with fat suppression images reveal enhanced synovium in the anterior recess (solid arrowheads) and posterior recess (open arrowheads). Both readers assigned a synovial visibility grade of 4 (fully visible synovium with good tissue contrast) in all joint compartments. Both readers assigned a synovial thickness score of 0 (maximum thickness < 2 mm) in all joint compartments. (**c**,**d**) Corresponding axial fluid-attenuated inversion recovery sequence with fat suppression (FLAIR-FS) images in the same level demonstrate hyperintense synovium at the location corresponding with CE-T1 images. Note that synovium appears thicker with better tissue contrast compared to CE-T1 images. Both readers assigned a synovial visibility grade of 4 (fully visible synovium with good tissue contrast) in all joint compartments. For synovial thickness score, reader 1 assigned 0, 0, 0, and 1 (maximum thickness 2–4 mm) and reader 2 assigned 1, 0, 0, and 1 in the anterior recess, anteromedial gutter, anterolateral gutter, and posterior recess, respectively.

The synovium appeared as a hyperintense region lining the ankle joint on FLAIR-FS images (Figure 3, Figure 4 and Figure 5). On both FLAIR-FS and CE-T1 images, the synovium was fully visible with good tissue contrast in most joint compartments, with mean visibility grades above 3.7 for both readers (Table 2). When comparing synovial visibility grades in each joint compartment, all but the posterior recess by reader 1 did not show significant differences between FLAIR-FS and CE-T1 images. The average synovial visibility grade of the whole compartment was lower on FLAIR-FS images than on CE-T1 images (reader 1, 3.88 ± 0.34 vs. 3.93 ± 0.26, *p* = 0.016; reader 2, 3.92 ± 0.32 vs. 3.97 ± 0.20, *p* = 0.009). However, when synovial visibility grades were dichotomized into partial visibility (grades 1 and 2) and full visibility (grades 3 and 4), the grades were not significantly different between FLAIR-FS and CE-T1 images (reader 1, *p* = 1.000; reader 2, *p* = 0.688) (Table 3). 

Data are presented as the number of joint compartments (percentage). Synovial visibility was graded as follows: grade 1, no visible synovium; grade 2, partially visible synovium; grade 3, fully visible synovium with low tissue contrast; and grade 4, fully visible synovium with good tissue contrast. *p* values were determined using the McNemar test.

FLAIR-FS = fluid-attenuated inversion recovery sequence with fat suppression, CE-T1 = contrast-enhanced, T1-weighted sequence.

### 3.3. Comparison of Synovial Thickness on FLAIR-FS and CE-T1 Images

Enhancing synovium on CE-T1 images often appeared as two separate layers on FLAIR-FS images with intervening dark signal intensity foci, which were suppressed signals from the joint fluid (Figure 3). The synovium barely appeared thicker and more prominent on FLAIR-FS images than on CE-T1 images (Figure 4). Consistent with these findings, synovial thickness scores were significantly lower in FLAIR-FS images compared to CE-T1 images regardless of joint compartments (summed synovial thickness score, 1.98 ± 2.19 vs. 2.77 ± 2.42 for reader 1 and 1.63 ± 1.97 vs. 3.06 ± 2.65 for reader 2, both *p* < 0.001). 

The agreement in synovial thickness scores between the FLAIR-FS and CE-T1 images was moderate to substantial (reader 1, κ = 0.65; reader 2, κ = 0.41) (Table 4). The agreement of summed synovial thickness scores in the four joint compartments was moderate to excellent (reader 1, ICC = 0.90; reader 2, ICC = 0.72). 

Data are presented as the number of joint compartments (percentage). Data in brackets are 95% confidence intervals. Synovial thickness was scored semi-quantitatively according to the maximum thickness in each compartment. Weighted kappa statistics were used to determine the κ values.

FLAIR-FS = fluid-attenuated inversion recovery sequence with fat suppression, CE-T1 = contrast-enhanced, T1-weighted sequence. 

### 3.4. Interobserver and Intraobserver Reliability in the Assessment of Synovium

The interobserver agreement for synovial visibility on the four-point scale was fair for both FLAIR-FS and CE-T1 images (FLAIR-FS, κ = 0.32; CE-T1, κ = 0.27). When synovial visibility grades were dichotomized into partial visibility (grades 1 and 2) and full visibility (grades 3 and 4), the interobserver agreement for synovial visibility was moderate for both FLAIR-FS and CE-T1 images (FLAIR-FS, κ = 0.57; CE-T1, κ = 0.50). For synovial thickness, the interobserver agreement was moderate to substantial (FLAIR-FS, κ = 0.54; CE-T1, κ = 0.74) for synovial thickness scores and good to excellent (FLAIR-FS, ICC = 0.86; CE-T1, ICC = 0.95) for summed synovial thickness scores. 

The intraobserver agreement for synovial visibility was moderate to substantial for both readers (reader 1, κ = 0.59 for FLAIR-FS, κ = 0.76 for CE-T1; reader 2, κ = 0.67 for FLAIR-FS, κ = 0.51 for CE-T1). The intraobserver agreement for synovial thickness was substantial for both readers (reader 1, κ = 0.64 for FLAIR-FS, κ = 0.68 for CE-T1; reader 2, κ = 0.68 for FLAIR-FS, κ = 0.78 for CE-T1). The intraobserver agreement for the summed synovial thickness scores was excellent for both readers (reader 1, ICC = 0.92 for FLAIR-FS, ICC = 0.93 for CE-T1; reader 2, ICC = 0.93 for FLAIR-FS, ICC = 0.95 for CE-T1).

## 4. Discussion

Our study demonstrated the feasibility of FLAIR-FS for the assessment of ankle synovitis without contrast enhancement. The synovium was fully visible in almost every joint compartment and well differentiated from the joint fluid on FLAIR-FS images. Although the average synovial visibility on FLAIR-FS images was lower than that on CE-T1 images, dichotomized synovial visibility grades were not significantly different between FLAIR-FS and CE-T1 images. The synovial thickness on FLAIR-FS showed good agreement with that on CE-T1. In addition, FLAIR-FS images adequately depicted two separate layers of the synovium when CE-T1 images could not. The results of our study indicate that FLAIR-FS is a feasible non-contrast sequence for the assessment of ankle synovitis.

A variety of non-contrast MRI sequences have been suggested for the assessment of synovitis, given the disadvantages of contrast media injection. Diffusion-weighted imaging was introduced as a reliable method to detect synovitis in juvenile idiopathic arthritis using different diffusional characteristics of the synovium and joint fluid [17,18,19]. However, assessment of synovitis was limited to subjective interpretation of the experienced radiologists or quantitative measurement of apparent diffusion coefficient values because the resolution of diffusion-weighted imaging was low. Quantitative double echo in steady-state imaging, which has a higher resolution than diffusion-weighted imaging, displayed synovitis with good diagnostic performance in knee osteoarthritis [20]. However, additional image processing was required to produce images showing synovitis. Recently, several MRI sequences that combine fat and water suppression techniques have been reported to be efficient in visualizing synovitis. A double inversion recovery sequence, which uses two inversion times for both fat and water suppression, was applied for the assessment of knee synovitis and showed a good correlation with CE-T1 [21,22]. Fluid-attenuated inversion recovery sequence (FLAIR) was combined with spectral presaturation inversion recovery or chemically selective fat suppression, showing high diagnostic accuracy in the evaluation of synovitis in the hip or knee [10,11]. In this study, we applied FLAIR and chemically selective fat suppression to evaluate ankle synovitis. This is the first study to demonstrate efficacy of a non-contrast MRI sequence in the assessment of synovitis in the ankle joint.

In the current study, the average synovial visibility on FLAIR-FS images was lower than that on CE-T1 images. This is likely due to the relatively lower signal-to-noise ratio of FLAIR caused by the use of inversion pulse. It is hypothesized that the image quality and signal-to-noise ratio could be improved in FLAIR-FS by incorporating denoising techniques. Additionally, increasing the number of averages would improve the signal-to-noise ratio, thus enhancing synovial visibility. Further research is warranted to improve the image quality and signal-to-noise ratio of FLAIR-FS.

We observed that the synovium commonly appeared thinner on FLAIR-FS images than on CE-T1 images. A definite advantage of CE-T1 over non-contrast MRI sequences is that the synovium is effectively differentiated from joint fluid because only inflamed synovium would show enhancement. However, diffusion of the contrast material into the joint fluid may cause overestimation of the synovium on CE-T1 at a late phase [23,24]. According to the review article by Steinbach et al. [25], this phenomenon was clearly demonstrated in small joints such as the ankle, where diffusion took only 5–10 min after the injection of contrast because joint fluid and the synovium are in proximity. In contrast, significant diffusion took more than 10 min in the knee joint, which has a large distance between the synovium and joint fluid [23,25]. Therefore, we speculated that FLAIR-FS can reveal the more accurate extent of the synovium than CE-T1, especially in small joints such as the ankle, compared to large joints such as the knee and hip. Consistent with our speculation, the dark signal intensity of the joint fluid on FLAIR-FS made it possible to distinguish the synovium and joint fluid. In contrast, two separate layers of synovium and intervening joint fluid were sometimes indistinguishable on CE-T1 images and appeared as a layer of thick synovium, probably owing to diffusion of the contrast material. Similar to our results, Treutlein et al. reported that FLAIR-FS underestimated the amount of synovitis compared to CE-T1 on a 7T MRI [12]. 

On the contrary, we rarely observed that the synovium appeared thicker and more prominent on FLAIR-FS than on CE-T1 images. A possible reason for this phenomenon is that the low spatial resolution of FLAIR-FS compared to that of CE-T1 resulted in an indistinct margin of the synovium on FLAIR-FS images. According to the study conducted by Son et al. [21], synovial thickness of the knee on the double inversion recovery sequence was consistently greater than that on the CE-T1. They attributed their results to the reduced resolution and sharpness of the double inversion recovery images. However, only a limited number of cases in our study showed thicker synovium on FLAIR-FS than on CE-T1. The different results might be because diffusion of the contrast into joint fluid is faster in the ankle than in the knee. Another explanation could be that FLAIR-FS demonstrated non-enhancing chronic synovial hypertrophy, while CE-T1 did not. Yoo et al. [10] reported that FLAIR-FS depicted non-enhancing chronic synovitis, which was also observed on proton density-weighted sequences. However, we could not observe synovium on the corresponding T2-weighted images in cases showing thicker synovium on FLAIR-FS than on CE-T1. This might be due to the small capacity of the ankle joint and the low sensitivity of T2-weighted images showing synovitis. 

FLAIR-FS can reduce the cost of contrast administration and is safe because it does not require the use of contrast agents. FLAIR-FS can be especially helpful in evaluating ankle synovitis in patients who are unable to undergo contrast-enhanced MRI for various reasons. Although gadolinium-based contrast agents are generally considered safe, contrast administration should be avoided depending on the patient’s medical condition. Firstly, patients with previous moderate to severe allergic reactions to gadolinium-based contrast agents should avoid contrast media injection [26]. Additionally, since nephrogenic systemic fibrosis rarely occurs in patients with chronic kidney disease or acute kidney injury [26,27], the use of contrast agent should be restricted in this patient population. Lastly, gadolinium contrast agents are generally advised against in pregnant women due to potential risks to the fetus [26]. In these patient populations, FLAIR-FS can serve as a good alternative to CE-T1 when the diagnosis of synovitis is necessary. Future study is required to investigate the correlation between FLAIR-FS and CE-T1 in evaluating synovitis in these specific patient populations.

Nevertheless, it may be more appropriate to evaluate synovitis using CE-T1 rather than FLAIR-FS in certain circumstances. First, CE-T1 is more appropriate for assessing the activity of synovitis than FLAIR-FS. In FLAIR-FS, both active synovitis and non-enhancing chronic synovial fibrosis is visualized [10]. Although it is helpful for evaluating the overall extent of synovitis, distinguishing active synovitis from chronic fibrosis can be challenging on FLAIR-FS. In addition, CE-T1 would be more accurate in evaluating treatment response for synovitis for the same reason. Therefore, it is necessary to properly select the appropriate sequence based on the patient’s clinical situation.

Chemically selective fat suppression was used for fat suppression in our study because we adopted the FLAIR-FS technique used in previous studies [10,12]. This was to investigate the feasibility of the previously reported FLAIR-FS technique for ankle joints. However, chemically selective fat suppression is prone to heterogeneous fat suppression owing to B0 and B1 heterogeneity [28,29]. Recently, the Dixon technique has been widely used for fat suppression in musculoskeletal MRI because of several advantages. The Dixon technique provides homogeneous and efficient fat suppression even with metal implants because it is robust to B0 and B1 heterogeneity [28,29]. In addition, Dixon sequences have a higher signal-to-noise ratio than inversion recovery pulse sequences [28,29]. Therefore, we expect that the FLAIR-FS technique applying Dixon fat suppression can provide images with superior quality compared to the FLAIR-FS technique using chemically selective fat suppression, double inversion recovery, or spectral presaturation inversion recovery. Further study using the Dixon technique is warranted.

This study had several limitations. First, histologic correlation was not performed in this study. Further cadaveric or arthroscopic studies with histologic correlations are needed to confirm the exact thickness of the synovium. Second, the lower resolution and signal-to-noise ratio of FLAIR-FS compared to those of CE-T1 might have affected the evaluation of synovitis on FLAIR-FS. However, we believe this limitation would be acceptable because synovial visibility and thickness on FLAIR-FS showed good agreement with those on CE-T1. Third, we did not assess the diagnostic performance of FLAIR-FS for detecting synovitis using CE-T1 as a reference standard. This was because chronicity and activity of synovitis, as well as diffusion of the contrast material into the joint fluid, might have affected the thickness of the synovium on CE-T1 images. Fourth, incomplete fat suppression by the chemically selective fat suppression technique might have affected the evaluation of synovitis. However, the results of comparisons between FLAIR-FS and CE-T1 are unlikely to be affected since both sequences used the same fat suppression technique. Finally, the study population was heterogeneous, and normal controls were not included owing to the retrospective design of the study. However, it is expected that the inclusion of joint compartments demonstrating varying degrees of synovitis would minimize selection bias. Additionally, approximately half of the joint compartments included in the study exhibited a normal synovial thickness of 2 mm or less. Further study with prospective design is warranted.

## 5. Conclusions

In conclusion, FLAIR-FS is a feasible MRI sequence for the evaluation of ankle synovitis without contrast enhancement. FLAIR-FS may reveal the extent of the ankle synovium more accurately than CE-T1 without diffusion of a contrast material into the joint fluid. In actual clinical practice, FLAIR-FS can be a good alternative to CE-T1 in evaluating ankle synovitis, particularly in patients who cannot use contrast agents.

## Figures and Tables

**Table 1:** Imaging parameters of FLAIR-FS and CE-T1 images.

Parameters	FLAIR-FS	CE-T1
TR/TE (ms)	9000/93	306–871/10
Inversion time (ms)	2100	N/A
Flip angle (°)	150	120
Echo train length	22	3
Bandwidth (kHz/pixel)	269	265
Field of view (mm)	140	140
Matrix size	320 × 154	320 × 182
Pixel size (mm)	0.44 × 0.91	0.44 × 0.77
Slice thickness/gap (mm)	3/0	3/0
Number of signals acquired	1	2
Number of sections	36	36
Fat suppression	Chemically selective	Chemically selective
Acquisition time (min)	2:42	3:17

FLAIR-FS = fluid-attenuated inversion recovery sequence with fat suppression, CE-T1 = contrast-enhanced, T1-weighted sequence, TR = repetition time, TE = echo time, and N/A = not applicable.

**Table 2:** Comparison of FLAIR-FS and CE-T1 for the assessment of synovitis.

	Synovial Visibility Grade			Synovial Thickness Score		
	FLAIR-FS	CE-T1	*p* Value	FLAIR-FS	CE-T1	*p* Value
**Reader 1**						
Anterior	3.89 ± 0.34	3.97 ± 0.18	0.052	0.59 ± 0.72	0.88 ± 0.81	<0.001 *
Anteromedial	3.87 ± 0.37	3.90 ± 0.33	0.491	0.32 ± 0.61	0.40 ± 0.64	0.046 *
Anterolateral	3.98 ± 0.15	3.99 ± 0.10	0.564	0.50 ± 0.67	0.69 ± 0.73	0.001 *
Posterior	3.78 ± 0.42	3.88 ± 0.32	0.041 *	0.57 ± 0.74	0.79 ± 0.80	<0.001 *
Whole joint	3.88 ± 0.34	3.93 ± 0.26	0.016 *	1.98 ± 2.19	2.77 ± 2.42	<0.001 *
**Reader 2**						
Anterior	3.96 ± 0.25	3.98 ± 0.21	0.577	0.52 ± 0.68	0.94 ± 0.87	<0.001 *
Anteromedial	3.86 ± 0.43	3.96 ± 0.29	0.050	0.32 ± 0.63	0.57 ± 0.78	<0.001 *
Anterolateral	3.95 ± 0.27	3.99 ± 0.10	0.157	0.32 ± 0.63	0.67 ± 0.79	<0.001 *
Posterior	3.91 ± 0.28	3.97 ± 0.18	0.132	0.47 ± 0.71	0.88 ± 0.88	<0.001 *
Whole joint	3.92 ± 0.32	3.97 ± 0.20	0.009 *	1.63 ± 1.97	3.06 ± 2.65	<0.001 *

Data are presented as mean ± standard deviation. *p* values were determined using the Wilcoxon signed-rank test. *p* values with an asterisk (*) are statistically significant. Synovial visibility was graded as follows: grade 1, no visible synovium; grade 2, partially visible synovium; grade 3, fully visible synovium with low tissue contrast; and grade 4, fully visible synovium with good tissue contrast. Synovial thickness was scored semi-quantitatively according to the maximum thickness in each compartment, as follows: score 0, <2 mm; score 1, 2–4 mm; and score 2, ≥4 mm.

**Table 3:** Dichotomized synovial visibility graded on FLAIR-FS and CE-T1 images.

Synovial Visibility	Partially Visible		Fully Visible		*p* Value
	Grade 1	Grade 2	Grade 3	Grade 4	
**Reader 1**					
FLAIR-FS	0 (0.0%)	2 (0.5%)	41 (10.9%)	333 (88.6%)	1.000
CE-T1	0 (0.0%)	1 (0.3%)	22 (5.9%)	353 (93.9%)	
**Reader 2**					
FLAIR-FS	0 (0.0%)	5 (1.3%)	20 (5.3%)	351 (93.4%)	0.688
CE-T1	0 (0.0%)	3 (0.8%)	4 (1.1%)	369 (98.1%)	

Mean ± standard deviation of the summed synovial thickness score in four joint compartments (score of 0–8). FLAIR-FS = fluid-attenuated inversion recovery sequence with fat suppression, CE-T1 = contrast-enhanced, T1-weighted sequence.

**Table 4:** Synovial thickness scored on FLAIR-FS and CE-T1 images in each joint compartment.

Synovial Thickness	Score 0 (<2 mm)	Score 1 (2–4 mm)	Score 2 (≥4 mm)	κ Value
**Reader 1**				
FLAIR-FS	233 (62.0%)	100 (26.6%)	43 (11.4%)	0.65 [0.56, 0.71]
CE-T1	187 (49.7%)	118 (31.4%)	71 (18.9%)	
**Reader 2**				
FLAIR-FS	261 (69.4%)	77 (20.5%)	38 (10.1%)	0.41 [0.34, 0.49]
CE-T1	187 (49.7%)	90 (23.9%)	99 (26.3%)	

## Data Availability

The data presented in this study are available upon request from the corresponding author.

## Supplementary Materials

The following supporting information can be downloaded at: https://www.mdpi.com/article/10.3390/diagnostics13111960/s1, Supplemental Table S1: Mean signal intensity of the joint fluid with different inversion time.
