# Supplementary material for: Fluid-Attenuated Inversion Recovery Sequence with Fat Suppression for Assessment of Ankle Synovitis without Contrast Enhancement: Comparison with Contrast-Enhanced MRI
Source: Diagnostics (Basel). 2023 Jun 4;13(11):1960. doi: 10.3390/diagnostics13111960 (PMC10252366; doi:10.3390/diagnostics13111960)
Supplement: Supplementary file 1 [file diagnostics-13-01960-s001.zip › diagnostics-2391362-supplementary.pdf]

Supplemental Table S1. Mean signal intensity of the joint fluid with different inversion time

Inversion time	Signal intensity of the joint fluid
2000 ms	31.8 ± 11.6
2100 ms	17.8 ± 9.2
2200 ms	34.5 ± 11.0

Data are presented as mean ± standard deviation. To determine the optimal inversion time for FLAIR-FS, which effectively nulls joint fluid signals, several test scans were performed using inversion times between 2000 and 2200 ms. Consequently, an inversion time of 2100 ms was selected.
